# Understanding the Canadian adult CT head rule trial: use of the theoretical domains framework for process evaluation

**DOI:** 10.1186/1748-5908-8-25

**Published:** 2013-02-21

**Authors:** Janet A Curran, Jamie Brehaut, Andrea M Patey, Martin Osmond, Ian Stiell, Jeremy M Grimshaw

**Affiliations:** 1 Clinical Epidemiology Program, Ottawa Hospital Research Institute, Civic Campus, Ottawa, ON, Canada; 2 Children’s Hospital of Eastern Ontario Research Institute, Ottawa, ON, Canada; 3 Department of Pediatrics, University of Ottawa, Ottawa, ON, Canada; 4 Department of Emergency Medicine, University of Ottawa, Ottawa, ON, Canada; 5 Department of Medicine, University of Ottawa, Ottawa, ON, Canada

## Abstract

**Background:**

The Canadian CT Head Rule was prospectively derived and validated to assist clinicians with diagnostic decision-making regarding the use of computed tomography (CT) in adult patients with minor head injury. A recent intervention trial failed to demonstrate a decrease in the rate of head CTs following implementation of the rule in Canadian emergency departments. Yet, the same intervention, which included a one-hour educational session and reminders at the point of requisition, was successful in reducing cervical spine imaging rates in the same emergency departments. The reason for the varied effect of the intervention across these two behaviours is unclear. There is an increasing appreciation for the use of theory to conduct process evaluations to better understand how strategies are linked with outcomes in implementation trials. The Theoretical Domains Framework (TDF) has been used to explore health professional behaviour and to design behaviour change interventions but, to date, has not been used to guide a theory-based process evaluation. In this proof of concept study, we explored whether the TDF could be used to guide a retrospective process evaluation to better understand emergency physicians’ responses to the interventions employed in the Canadian CT Head Rule trial.

**Methods:**

A semi-structured interview guide, based on the 12 domains from the TDF, was used to conduct telephone interviews with project leads and physician participants from the intervention sites in the Canadian CT Head Rule trial. Two reviewers independently coded the anonymised interview transcripts using the TDF as a coding framework. Relevant domains were identified by: the presence of conflicting beliefs within a domain; the frequency of beliefs; and the likely strength of the impact of a belief on the behaviour.

**Results:**

Eight physicians from four of the intervention sites in the Canadian CT Head Rule trial participated in the interviews. Barriers likely to assist with understanding physicians’ responses to the intervention in the trial were identified in six of the theoretical domains: beliefs about consequences; beliefs about capabilities; behavioural regulation; memory, attention and decision processes; environmental context and resources; and social influences*.* Despite knowledge that the Canadian CT Head Rule was highly sensitive and reliable for identifying clinically important brain injuries and strong beliefs about the benefits for using the rule, a number of barriers were identified that may have prevented physicians from consistently applying the rule.

**Conclusion:**

This proof of concept study demonstrates the use of the TDF as a guiding framework to design a retrospective theory-based process evaluation. There is a need for further development and testing of methods for using the TDF to guide theory-based process evaluations running alongside behaviour change intervention trials.

## Background

Clinical decision rules are developed using rigorous methodology to assist clinicians with decision-making in specific therapeutic and diagnostic situations [1,2]. The rules generally identify the specific components of a patient’s history, physical exam, and laboratory tests that are relevant for diagnostic or therapeutic decision-making [3]. Clinical decision rules can be particularly useful in emergency practice environments, which are characterised by diverse and unpredictable patient presentation and a focus on managing ‘patient flow.’ Patient flow in the emergency department refers to the passage of time from entry in the front door through to discharge out of the department, also described as input-throughput-output [4]. High quality tools such as clinical decision rules can improve efficiency in decision-making and have the potential to improve quality of care.

Minor head injury is a frequent presentation in the emergency department, and studies demonstrate wide variation in the use of computed tomography (CT) to identify clinically important brain injury [5]. The Canadian CT Head Rule was prospectively derived [6] and validated [7] to assist clinicians with diagnostic decision-making in adult patients with minor head injury. The rule identifies five high-risk criteria for neurosurgical intervention and two medium-risk criteria for detecting brain injury on CT [8](See Canadian CT Head Rule in Additional file 1). The rule has demonstrated high sensitivity (100%; 95% CI 91to 100) and reliability for identifying clinically important brain injuries. If used appropriately by emergency physicians, the rule has the potential to reduce the number of unnecessary CT scans without jeopardizing care [9].

In a recent matched-pair cluster-randomized trial, a series of simple and inexpensive implementation strategies failed to reduce CT scanning rates in participating emergency departments [10]. In fact, CT imaging rates increased from the ‘before’ to the ‘after’ period in both the control (67.5% to 74.1%, absolute difference +6.7%) and intervention (62.8% to 76.2%, absolute difference +13.3%) sites. Further, physicians misinterpreted the rule in 17.5% of the cases, ordering CT imaging in 141 of 909 cases, despite contrary recommendations according to the rule. Yet, the same series of simple and inexpensive strategies led to successful implementation of the Canadian C-Spine Rule in the same emergency departments, resulting in a relative reduction of 12.8% (61.7% versus 53.3%) in the diagnostic imaging rate of cervical spines [11]. The intervention design used in both trials was based on theoretical considerations of behaviour change, available evidence, and consultation with study collaborators [12]. The strategies, which were intended to target different barriers at the individual and system level, included establishing local consensus, a one-hour educational session, and a mandatory reminder at the point of requisition. The reason behind the variation in effect across the two studies remains unclear.

The effects of an intervention may vary by patient condition or environmental context as the causal mechanisms are often modified by different enablers and barriers [13].Theory-based process evaluations, which collect data on theoretical constructs alongside a trial, can provide insight into the causal mechanisms and effect modifiers of an intervention [14]. However, in the absence of real-time fieldwork data, a theory-oriented post trial evaluation can serve as an important source of data for understanding what happened in the trial and possibly refining an intervention for the future [15]. Retrospective theory-based process evaluations have provided useful information for interpreting results in implementation trials of a structured recall and prompting intervention [16], and enhanced feedback and brief educational reminders [17].

Identifying the most appropriate theory to guide a theory-based process evaluation from the wide array of available behaviour change theories can be daunting. The Theoretical Domains Framework (TDF) integrates 33 behaviour change theories and 128 explanatory constructs into a more accessible structure consisting of 12 theoretical domains. [18]. To date, the TDF has been used to identify barriers for health behaviour change [19-21] and to guide design of behaviour change strategies [22-24]. Our study was a proof of concept study to operationalize the TDF to conduct a retrospective theory-based process evaluation. More specifically, we wanted to determine whether the domains in the TDF could help explain participants’ lack of response to the strategies employed in the Canadian CT Head Rule trial. This article is one in a series of articles documenting the development and use of the Theoretical Domains Framework (TDF) to advance the science of implementation research. The Series’ introductory article [25] provides an overview of the articles contained in the TDF Series.

## Methods

### Design

This was a qualitative study with physicians from the six intervention sites who participated in the Canadian CT Head Rule trial [10].

### Participants

In the absence of any real-time process evaluation data, the learning and experiences of program leaders and trial participants can serve as an important source of post hoc data [15]. Across the six intervention sites, 150 physicians were involved in implementing the Canadian CT Head Rule. Because we were interested in understanding physician response to the implementation strategies employed in the Canadian CT Head Rule trial, we used a purposive sampling technique to specifically recruit the project leads and physicians from the six intervention sites who participated in the study. Physicians from the six intervention sites who were not working in the ED during the time of the trial were excluded.

### Materials

An interview guide was developed based on the 12 domains included in the TDF (knowledge; skills; social/professional role and identity; beliefs about capabilities; beliefs about consequences; motivation and goals; memory, attention and decision processes; environmental context and resources; social influences; emotion; behavioural regulation; nature of the behaviours). We developed two to five questions per domain to cover the range of constructs assigned to each domain. Additional prompts were prepared to probe domains if further clarification was needed. Each of the questions in the interview guide focused on the behaviour of interest – physicians’ use of the Canadian CT Head Rule to man age adult patients who present to the emergency department with minor head injury. For example, to explore the influence of *social/professional role and identity,* the following question was asked: ‘Is there anything about belonging to a professional group of emergency physicians that influences how you use the Canadian CT Head Rule to manage adults who present to the emergency department with minor head injury?’ This interviewing strategy ensured participants spoke about experiences pertinent to use of the Canadian CT Head Rule. Demographic questions were included to capture information about participants’ clinical and specialty training in emergency medicine. An emergency physician (MO), a cognitive psychologist (JB) and a health behavior researcher (RI) reviewed the interview schedule for face and content validity. Wording was further refined to reduce repetition and enhance clarity following a pilot interview (Additional file 2). Although 12 domains are identified in the TDF, questions in the twelfth domain, nature of the behaviours, are intended to describe the characteristics of the behaviour of interest. Therefore, we will report on the possible barriers identified across 11 domains and use data gathered under domain 12 to describe how the behaviour was carried out in practice.

### Procedure

A letter explaining the purpose of the study was sent to the project leads at each of the intervention sites (three academic, three community) from the Canadian CT Head Rule trial. Two sites (one academic, one community) failed to respond despite three reminders. Project leads who agreed to participate were asked to provide contact information for the physicians in their centre who participated in the original study. Letters of invitation were emailed to 32 physicians who met inclusion criteria from the four intervention sites. Physicians were asked to sign consent prior to being interviewed and were offered a $50 honorarium for their participation. Telephone interviews were arranged at a time that was convenient for the participant and were digitally recorded. Telephone interviews can be used productively in qualitative studies particularly when the research focus is narrow and immersion by the researcher in the environment is not requisite [26,27]. In our study, telephone interviewing was particularly useful to reach geographically dispersed and busy emergency clinicians. The recordings were transcribed and anonymised. All interviews were carried out by one interviewer (JC) and lasted between 20 and 40 min (M = 28.45; SD = 6.10). The study was approved by the Research Ethics Board of the Ottawa Hospital.

### Analysis

The analytical method involved an iterative process of data collection and analysis [28] and two independent coders. All transcripts were coded in NVivo 8 [29]. We used a directional approach [30] to content analysis in order to systematically categorize the textual data into domains. Two reviewers (JC, AP), working independently, completed the coding, moving back and forth between the transcripts and the theoretical domains in the TDF. The reviewers met after coding the first two transcripts to compare results. Coding differences were resolved through discussion. The remaining six transcripts were subsequently coded as the interviews were completed using the same approach. When all coding was completed, reviewers compared results and used discussion to resolve coding differences. One reviewer (JC) generated a list of specific beliefs from the utterances coded in each domain, and the list was subsequently confirmed by a second reviewer (AP). Two reviewers (JC, AP) used discussion to determine which domains might help explain physicians’ responses to the intervention. Domains likely to explain use of the Canadian CT Head rule were identified through consideration of: the presence of conflicting beliefs within a domain that would signal variation in provider attitudes and beliefs; the frequency of specific beliefs across transcripts; and the likely strength of the impact of a belief on the behaviour.

## Results

Eight physicians from four of the intervention sites agreed to take part in the interviews. Three of the physicians were project leads for the trial in their emergency departments. Physicians had been practicing in an emergency department for between seven and thirty years (mean = 15.5), and six had specialty or sub-specialty training in emergency medicine. When asked to comment on the strategies used in the Canadian CT Head Rule trial, all physicians identified reminders, particularly mandatory completion of the study form at the point of CT scan requisition, as the most useful strategy to encourage use of the rule (‘mandating that the CT Head will not be done unless the form is filled in, that is probably the best way to do it because then you have to do it before you get the test done’ [Interview 4], ‘the mandated x-ray requisition was probably the most useful’ [Interview 8]).

The behaviour of interest in this study was use of the Canadian CT Head Rule to manage adults who present to the emergency department with minor head injury. Table 1 outlines 29 beliefs, which were identified in 11 theoretical domains. The number of participants who expressed the belief is presented in the final column. It is interesting to note that, in general, when physicians were asked specific questions about how they used the Canadian CT Head rule in their practice, they often didn’t speak about the rule itself but actually talked about times when they did or did not order a CT: as if the behaviour – use of the rule – was equal to not ordering a CT. This might suggest that different physicians perceive use of the rule in different ways and that the behaviour may be complicated by these perceptions.

**Table 1:** Summary of domains and specific beliefs

**Domains LIKELY to explain response to implementation of the Canadian CT Head Rule**			
Domain	Specific Belief	Sample Quote	Frequency
Beliefs about capabilities	I am confident I can apply the Canadian CT Head rule	‘quite confident, strongly confident’ #6;	5/8
		‘I think it is very easy’ #1;	
		‘quite confident’ #7	
	*Some of the criteria are not always clear for me	‘the mechanism which is sometimes a little confusing, the fall from elevation always confuses me as well as some of my colleagues’#1; ‘dangerous mechanisms I find that’s a bit vague sometimes’ #2; ‘over the age of 65 which is always a contentious one’ #3	4/8
	*It is challenging/easy to follow the rule when the department is busy	‘I go and see the patient and they’ve already had a CT’#4; ‘even when we’re busy I think it’s something that could be done relatively briefly’ #7; ‘when I got too many patients and I am trying to thin things out I just can’t remember all the criteria’ #2	4/8
Beliefs about consequences	Use of the CT head rule supports my decision making	‘your decision making is validated by a clinical decision rule’ #5; ‘it gives you some confidence in your clinical decision-making’ #1; ‘helps me decide whether I need to accept a transfer’ #8	5/8
	Use of the CT head rule decreases radiation exposure for patients	‘improves the radiation per patient’ #2; ‘needless radiation’ #3; ‘exposure to radiation is lower’ #4	5/8
	*Use of the CT head rule improves/hinders patient flow in the department	‘it improves flow in the emergency department’ #1;’It would increase favorably our patient flow’ #7; ‘every time you get a CT head it adds another hour or two to the length of stay’ #1	7/8
	The rule is used to explain to patients why they don’t need a head CT	‘often times we use it to explain to families why we’re not ordering a CT scan’ #3; ‘you can justify to them’ #5; ‘the first item is convincing patients they don’t need a CT’ #7	5/8
Behavioural regulations	*I rely on my own clinical judgment to guide my decision making when I am uncertain	‘concerned that they need a CT head, even though they don’t meet any of the criteria, I would still go ahead and do a CT head’ #2; ‘the category I take the most license with is the age category’ #8; ‘if I say “gee this isn’t making sense; I’m not comfortable” then I’ll do a CT scan’ #4	4/8
	*The rule is not easily accessible to me	‘wall charts in assessments rooms that make it easily accessible’ #7; ‘access to resources that allow you to reference quickly’ #5; ‘having it more in my face in my department’#2	7/8
	*Criteria in the Canadian CT Head Rule are flexible	‘we no longer use the rule, I mean that format’ #4;	4/8
		‘may have a few minor criteria that differ a little bit, they are pretty much all the same in my mind’ #2; ‘I think there is huge variation in how the rule is used’ #8	
Memory, attention, decision processes	*Criteria in the rule are easy/difficult to remember	‘I don’t know all the criteria off by heart’#2; ‘I probably remember most of them but there’s always one to two that I’ll miss’ #6	8/8
	Patient presentation cues my use of the rule	‘as soon as I see you know, bonked on head, transient loss of consciousness’ #4; ‘prime mechanism of injury triggers the use of the rule’ #3	8/8
	*I need to have the rule visible to remember to use it	‘when I do use the rule I have it in front of me’ #5; ‘so I want a piece of paper or you know something that will twig me’ #4	4/8
	*Reminders to use the rule would be helpful	‘we need to have a reminder’ #4; ‘a tool reminder would be most helpful’ #6	5/8
	*The number of criteria in the rule make it easy/difficult to remember	‘the limited number or short number of steps makes it easy’#1; ‘it’s too long unless you have it written down somewhere’ #2; ‘the rules are fairly brief and they don’t involve too many steps’ #7	5/8
Environmental context and resources	*The focus on patient flow influences my use of the rule	‘always under pressure to see more patients faster’ #2; ‘move them out the ED faster and free up a bed by getting a CT’ #8	5/8
	*Ease of access to the CT scan discourages use of the CT rule	‘more CT scans because they are more and more readily available’ #8; “we have ready access to CT 24 hrs a day’ #2; ‘it’s a bronze day with all these solar powered CT scanners in all the community hospitals’ #4	5/8
	*A busy department discourages/encourages use of the rule	‘being a busy ED it’s helpful to have one less thing to follow up on…probably encourages its use’ #3; ‘times when I have forgotten to use it when I’m just so busy’ #4; ‘when you are busy it’s hard to, you know, you have grounds to use it but it does slow you down’ #5	6/8
	*Patient presentation may bias use of the rule	‘by the time they get to us they are in a different state’ #5; ‘sometimes you are biased by the location of the patient and you forget it may be a minor head injury’ #6	4/8
Social influences	*Patients emotional state influences my use of the rule	‘the only influence that would change my mind is the patient’ #4; ‘there are times when I’ve stretched the rules a little bit or you know because of a patient’s anxiety’ #1;’ if they are highly anxious and really want a CT’ #2	5/8
	*Family’s emotions influences my use of the rule	‘at the younger age of the spectrum and there is a lot of family pressure or anxiety. It’s easy to get misguided by that’ #3	2/8
	Talking about the rule with residents or other learners reinforces use of the rule	‘you spit out the rules all the time with residents so it is just there’ #3;	4/8
		‘I go through the rule with referring physicians’ #6;	
		‘training physicians are always getting you to apply best practice’ #8	
**Domains UNLIKELY to explain response to implementation of Canadian CT Head Rule**			
Domain	Specific Belief	Sample quote	Frequency
Knowledge	The evidence that supports the rule is strong	‘it is a strong a piece of work as there is out there’ #4	7/8
		‘there is very strong evidence that it is a sensitive rule’#8;	
		‘I would rate it as very strong’#7	
Skills	You need experience as an emergency physician to use the rule	‘I think with some experience and some time they are easy to use’ #5;	5/8
		‘I think you need a fair bit of background to pick out subtleties’#2;	
		‘every doctor who is an emergency physician has the skill set to use it’ #4	
	It is simple to use	‘It is simple and can be used by pretty much anyone’ #3;	6/8
		‘the rule is simple as long as you have it in front of you’ #6	
Social/professional role and identity	Use of the rule is a professional standard in emergency medicine	‘has pretty much become the standard of care’ #7;	6/8
		‘your peers are doing it around the country’ #5;	
		‘I think it has become a standard of care’ #6;	
Motivation and goals	The Canadian CT Head rule is important	‘it is very important because it provides a framework to follow’ #3	6/8
		‘it’s a common presentation in our department and it’s a common referral we get from outside’ #6;	
		‘I am practicing current best evidence’ #8	
	Use of the Canadian CT Head Rule is compatible with my practice	‘Compatible because it’s a common presentation in our department, it’s a common referral we get from the outside as a referral centre so I think it’s a good tool to have’ #6;	7/8
		‘Absolutely yes compatible’ #7	
Emotion	Worry about missing an important injury is of concern in some situations	‘no clinical decision making rule is 100% so there is always a lingering worry’ #7; “the danger of missing a head injury is so high that people are just going ahead and ordering CT scans’ #8	3/8

*Specific beliefs likely to influence implementation of the Canadian CT head rule.

### Domains unlikely to explain physician response to implementation of the Canadian CT Head Rule

Knowledge about the Canadian CT Head Rule and awareness of the scientific rationale for the rule was high among study participants. A consistent comment across interviews was that a strong body of evidence supported the rule (seven responses). Participants also expressed knowledge of and confidence in the research team that developed the rule. When asked about skills needed to use the Canadian CT Head rule, participants reported that the rule was easy to use by emergency physicians with some experience (‘every doctor who is an emergency physician has the skill set to use it’ [Interview 4]). The rule was also identified as a professional standard in emergency practice (six responses). This suggests that social/professional role and identity was unlikely to pose a barrier to use of the rule. When asked how important they felt the rule was, most participants reported use of the rule as either important or very important (six responses) and compatible with their usual practice (‘compatible because it’s a common presentation in our department, it’s a common referral we get from the outside as a referral centre, so I think it’s a good tool to have’ [Interview 6]). This would suggest that the domain of *motivation and goals* was also not relevant in explaining physicians’ responses to the intervention of implementing the Canadian CT Head Rule trial. All physicians reported that, in general, using the rule did not create any emotional response (stress or anxiety) for them (‘No, I think if anything it actually reassures me’ [Interview 3]), which would suggest that emotion is unlikely to explain physicians’ responses to implementation of the rule.

### Domains likely to explain physician response to implementation of the Canadian CT Head Rule

Beliefs about consequences for using the rule generated a lot of discussion, with the majority of participants describing many benefits of using the rule. Participants reported that using the rule would generate positive outcomes for patients (‘exposure to radiation is lower’ [Interview 4]) and physicians (‘your decision making is validated by a clinical decision rule’ [Interview 5]). However, there was variation in their beliefs about how using the rule, which would influence their decision to order a CT, could impact patient flow in the department. This ranged from ‘every time you get a CT-head it adds another hour’ (Interview 1), thus hindering patient flow in the department, to ‘move them out of the ED faster and free up a bed by getting a CT’ (Interview 7), thus, ordering a CT would improve patient flow. Although all participants reported that the benefits of using the rule outweighed the risks, the conflicting beliefs expressed regarding impact of the rule on patient flow would suggest that beliefs in this domain might contribute to inconsistent use of the rule.

Participants were also mixed in their beliefs about capabilities to use the rule. While the majority reported a high level of confidence in using the rule, they also expressed a lack of confidence in interpreting some of the criteria in the rule, particularly mechanism of injury (‘the mechanism which is sometimes a little confusing, the fall from elevation always confuses me as well as some of my colleagues’ [Interview 1]). Challenges in using the rule were also reported in certain circumstances, for example, when the emergency department was busy (‘when I got too many patients and I am trying to think things out I just can’t remember all the criteria’ [Interview 2]) or when patient presentation was atypical. While all physicians reported that using the rule does not create any emotional response for them, when probed further, two physicians indicated that concern or worry about missing an important brain injury is relevant in some complex patient scenarios where the physicians’ intuition suggests scanning but the rule indicates not to scan (‘No clinical decision rule is 100% so there is always a lingering worry’ [Interview 7]).

Two issues surfaced as a consistent finding across all interviews when discussing questions related to memory, attention, and decision processes. First, respondents expressed some difficulty remembering the steps of the rule. The majority of physicians referenced the number of steps in a rule as an important factor, but they varied in their beliefs about whether the number of steps in the Canadian CT Head Rule made the rule easy or difficult to remember (‘the limited number or short number of steps makes it easy’ [Interview 1], ‘it’s too long unless you have it written down somewhere’ [Interview 2]). Second, respondents expressed problems remembering to use the rule. Although participants reported that patient presentation cued their use of the rule, many also suggested that having the rule visible was necessary to remember to use it.

A number of beliefs related to behavioural regulation also surfaced as potential barriers. Participants described a number of scenarios that would influence their use of the rule, including accessibility of the rule and the need for reminders to use the rule. It would also appear that some physicians do not use the Canadian CT Head rule as their only source to assist with decision-making in managing adults with minor head injury. Some talked about using more than one head rule or a mixture of steps from various rules in some instances (‘may have a few minor criteria that differ a little bit, they are pretty much all the same in my mind’ [Interview 2]). The rule is used as a guide for decision-making, but in situations where physicians are unsure if the patient fits the rule or they are concerned about how the rule is guiding their action, they would use caution and order a CT scan (‘concerned that they need a CT head, even though they don’t meet any of the criteria, I would still go ahead and do a CT head’ [Interview 2]).

When speaking about their environmental context and resources, physicians talked about how the physical and organizational context of emergency practice (*e.g.*, focus on patient flow, an overcrowded or busy department) can influence their use of the rule. ‘Times when I have forgotten to use it is when I’m just so busy’ (Interview 4). During a busy shift, use of the rule was also seen to either slow down or improve momentum and therefore could influence physicians’ use of the rule (‘when you are busy it`s hard to, you know, you have grounds to use it but it does slow you down’ [Interview 5]). The increasing availability of CT scans was also considered an important resource factor influencing use of the rule (‘more CT scans because they are more and more readily available’ [Interview 8]). The majority of participants suggested that with 24-hour access to CT scanners in most departments, the convenience and ease of obtaining other evidence to support their diagnosis is tempting. The social influences domain was also relevant to use of the Canadian CT Head Rule, particularly the influence of patient and family members (‘the only influence that would change my mind is the patient’ [Interview 4]). Physicians talked about how an anxious patient can influence them to order a CT even though the rule would indicate otherwise. This belief conflicts with the physicians’ concerns about unnecessary exposure to radiation; thus *social influences* would appear to act as barrier to the physicians’ use of the Canadian CT Head Rule in some situations.

## Discussion

Our study demonstrated that the TDF could provide a useful framework to guide a retrospective process evaluation from a theoretical perspective. Transcript analysis revealed a range of determinants that were likely to influence emergency physicians’ responses to the intervention of implementing the Canadian CT Head Rule. Six domains were identified that might pose barriers for use of the rule: beliefs about consequences; beliefs about capabilities; behavioural regulation; memory, attention, and decision processes; environmental context and resources; and social influences. It is worth noting that since the completion of this project, the structure of the TDF has been further refined through a three-step validation process and now includes 14 domains rather than 12 domains [31]. The refined framework has particular relevance for our study since two of the domains that were important in our study, beliefs about capabilities and beliefs about consequences, have been further separated into four distinct domains. It is possible that in future process evaluations, use of the refined framework could provide an even stronger explanatory basis for the results of intervention trials.

Physicians in our study were familiar with the Canadian CT Head Rule prior to the implementation trial because the same sites had also participated in the earlier derivation and validation trials, and those results had been presented at conferences and in journal publications. Despite knowledge that the Canadian CT Head Rule was highly sensitive and reliable for identifying clinically important brain injuries, and strong beliefs that the benefits for using the rule outweigh the risk, participants in our study reported that their use of the rule can vary under different patient and context scenarios.

The strategies employed in the Canadian CT Head Rule trial included a local consensus process, a single one-hour educational session with the distribution of pocket cards and posters, and a real time reminder at the point of requisition for the CT scan [10]. The educational session was intended to target physicians’ attitudes towards the rule. Physicians in our study believed the rule was valuable and that they possessed the requisite skills needed to use the rule under stable conditions. However, their beliefs about the consequences of using the rule and their capabilities to use the rule were unsteady, particularly when the department was busy or when patient presentation was not typical.

Physicians identified the reminder strategies as the most valuable strategy employed in the Canadian CT Head Rule Trial. However, memory, attention, and decision processes appeared to be linked with other relevant domains (behavioural regulation and environmental context and resources). Simple reminder strategies (posters, pocket cards, mandated requisition forms) such as those used in the Canadian CT Head Rule trial may not have fully addressed the complex nature of this potential barrier. These strategies might assist with remembering to use the rule under ideal conditions, but may not be useful in novel or complex patient presentations; thus they would not preclude clinicians from using the rule incorrectly (*i.e.*, adding steps or correctly interpreting the mechanism of injury criteria). The reminder strategies used were also unlikely to address clinicians’ beliefs about the benefits of using the rule when the department is busy. Previous studies of emergency physicians’ use of clinical decision rules report variation in the way a rule is used and applied [32]. Strategies such as action planning, barrier identification, or problem solving how to use the Canadian Head CT Rule in different patient scenarios might prove useful in future trials [33].

Elements related to the physical and organizational context, including patient and resource factors, such as those identified in this study are known to influence decision-making in emergency departments [34]. Emergency physicians are often required to manage multiple patients, with a diversity of presentations in a condensed time frame and many interruptions. Croskerry [35] suggests that the decision-making challenges in the emergency department are like no other clinical setting, with the ‘variety, novelty, distraction, and chaos, all juxtaposed to a need for expeditious and judicious thinking’ (p 720). These characteristics make emergency department environments prone to decision error [36]. Clinical decision rules simplify and increase the accuracy of clinicians’ diagnostic assessment [3]. They specify the smallest number of criteria from the history, physical assessment, and laboratory tests needed to make specific diagnostic decisions. However, the accuracy of a rule is dependent on clinicians’ consistently applying the rule exactly as it was derived and validated [32]. Some physicians in this study reported incorporating steps from other head rules into the Canadian CT Head Rule. Some also reported difficulty understanding and remembering all of the steps when using the rule. This would suggest that while clinicians report that they are using the rule, they may not be using it in the way it was intended to be used [37]. An educational strategy to improve general understanding of how to apply a clinical decision rule might be beneficial [38]. The conflict expressed regarding impact of the rule on patient flow when the emergency department is busy would suggest that a behaviour change intervention targeting outcome expectancies, a component construct in beliefs about consequences*,* might be useful.

Cognitive activity is the most important part of a clinician’s performance in the emergency department [36]. In an effort to manage multiple tasks in short time frames, clinicians will look for ways to conserve cognitive resources. Heuristic thinking tends to dominate clinical decision-making activities in this environment where uncertainty and narrow time frames are prevalent [39]. Clinicians reported using the rule to validate their decision-making and to explain to patients why they would not need a CT. Both of these scenarios might suggest that clinicians are applying the rule after they have already made their decision. When clinicians feel confident in their decision-making, they may be unaware of the correctness of their diagnosis and thus not open to using supports like clinical decision rules to change their decision [40]. In the case of minor head injuries, physicians may also employ the ‘rule out worst-case scenario’ strategy of decision making and order CTs for many patients to avoid missing a life-threatening diagnosis [41]. Fitting a clinical decision rule into the management of a clinical problem requires reflection on how clinicians make a diagnosis in a range of scenarios [38]. Decision-making process barriers may be addressed with behaviour change techniques, such as providing instruction on how to use the rule, or action planning strategies that support appropriate use of the Canadian CT Head rule under varied patient and context scenarios.

Increasing the appropriate use of the Canadian CT Head Rule will require attending to factors that influence that behaviour. A theory-based process evaluation, such as the one carried out in this project, can assist with explaining participant response to an intervention by highlighting behavioural determinants that continue to exist in the presence of a specific behaviour change intervention. A number of scholars have begun to outline strategies for mapping theoretical derived behavioral determinants to appropriate interventions [42,43]. Mapping behaviour change techniques to the relevant domains in our study would suggest that a complex intervention inclusive of techniques such as self-monitoring (beliefs about capabilities; beliefs about consequences; and memory, attention, and decision processes), persuasive communication or information about how to use clinical decision rules (*beliefs about consequences*), reminder strategies that target complex situations (memory, attention, and decision processes; behavioural regulation), and social processes of encouragement (social influences) may have led to a different outcome.

### Limitations

This study had a number of limitations. First, the response rate was low (eight individuals, four sites), so it is possible that we missed barriers unique to the non-participating intervention sites. This proof of concept study used a purposive sampling strategy to specifically target only those physicians from the intervention sites who participated in the Canadian CT Head Rule trial. The aim of our study was to identify domains that might assist with explaining physicians’ responses to the implementation strategies used in the trial; therefore, ensuring adequate population of the domains in the coding framework was an important factor to consider in determining data saturation [44]. Physicians and site champions from both academic and community practice settings were represented in our sample, and the barriers revealed using this theoretical approach offer some important insights about physicians’ responses to the interventions employed in the trial. Use of an iterative process for data collection and analysis allowed us to identify the point at which no new ideas were surfacing within the domains of the TDF and thus to feel confident that data saturation was achieved. Second, all participants identified use of the Canadian CT Head rule as a professional standard, so there is a potential that social desirability influenced their account of their experience in the trial. Participants were assured that their responses would be anonymised, and they were encouraged to expand on their experiences through additional probing questions. Third, participants were asked to give retrospective accounts of their experiences in the trial. Notwithstanding this limitation, common themes were identified across interviews and settings, which serve to corroborate individual experiences.

## Conclusion

The effectiveness of behaviour change interventions appears to vary across different clinical problems. An intervention, which included a one-hour educational session and reminders, was successful in reducing cervical spine imaging rates but failed to reduce CT imaging rates in the same set of emergency departments. In this proof of concept study, we used the TDF to conduct a retrospective process evaluation to better understand physicians’ responses to the interventions employed in the Canadian CT Head Rule trial. Our study findings demonstrate that the TDF can provide useful information about behavioural determinants that might aid in post-hoc interpretation of the results of a trial. We encourage researchers to further develop methods for using the TDF to guide theory-based process evaluations running alongside trials evaluating behaviour change interventions.

## Abbreviations

CT: Computed Tomography; TDF: Theoretical Domains Framework.

## Competing interests

IS was the Principle Investigator, and JMG was a co-investigator in the Canadian CT Head Rule trial. All other authors declare no competing interests. JMG is a member of the Editorial Board of Implementation Science.

## Authors’ contributions

JC (Lead Investigator) designed the study, conducted the interviews, led analysis and interpretation, and drafted the manuscript. JMG (Principal Investigator) oversaw the project and provided critical review of study design, study findings and manuscript development. JB provided critical review of study design and manuscript development. AP assisted with analysis, interpretation of study findings, and provided feedback on the manuscript. IS and MO provided feedback on study design and manuscript. All authors have read and approved final manuscript.

## Supplementary Material

Additional file 1 Canadian CT Head Rule. Click here for file

Additional file 2 Exploring the Canadian CT Head Rule Trial. Click here for file
