# Supplementary material for: Understanding the Canadian adult CT head rule trial: use of the theoretical domains framework for process evaluation
Source: Implement Sci. 2013 Feb 21;8:25. doi: 10.1186/1748-5908-8-25 (PMC3585785; doi:10.1186/1748-5908-8-25)
Supplement: Additional file 1 — Canadian CT Head Rule. [file 1748-5908-8-25-S1.pdf]

Canadian CT Head Rule

CT head is only required for minor head injury patients with any one of these findings:

High Risk (for Neurological Intervention)

1. GCS score < 15 at 2 hrs after injury
2. Suspected open or depressed skull fracture
3. Any sign of basal skull fracture*
4. Vomiting ≥ 2 episodes
5. Age ≥ 65 years

Medium Risk (for Brain Injury on CT)

6. Amnesia before impact ≥ 30 min
7. Dangerous mechanism ** *(pedestrian, occupant ejected, fall from elevation)*

***Signs of Basal Skull Fracture**

- hemotympanum, 'racoon' eyes, CSF otorrhea/ rhinorrhea, Battle's sign

**** Dangerous Mechanism**

- pedestrian struck by vehicle
- occupant ejected from motor vehicle
- fall from elevation ≥ 3 feet or 5 stairs

Rule Not Applicable If:

- Non-trauma cases
- GCS < 13
- Age < 16 years
- Coumadin or bleeding disorder
- Obvious open skull fracture

Stiell IG, et al. The Canadian CT Head Rule for Patients with Minor Head Injury. Lancet 2001;357:1391-96.
