# Supplementary material for: Understanding the Canadian adult CT head rule trial: use of the theoretical domains framework for process evaluation
Source: Implement Sci. 2013 Feb 21;8:25. doi: 10.1186/1748-5908-8-25 (PMC3585785; doi:10.1186/1748-5908-8-25)
Supplement: Additional file 2 — Exploring the Canadian CT Head Rule Trial. [file 1748-5908-8-25-S2.pdf]

Exploring the Canadian CT Head Rule Trial

Interview Schedule for Semi Structured Interview

Explanation

Thank you for agreeing to speak with me today. This interview should take approximately 20 to 30 minutes and will be audio taped to ensure that I accurately capture all of the key points that you share with me. Any identifying information (for example any individual health professional or patient names) that you use in the course of our discussion will be removed from the interview transcripts. If you wish to end the interview before I have asked all of the questions or if you wish to withdraw from the study at any time during the interview you are free to do so.

I would like to start by asking you a few questions about your practice

1. How many years have you worked in an emergency practice setting?
2. Do you have any speciality or subspecialty training?

Introductory Questions

1. Do you recall participating in the Canadian Adult CT Head Rule study that took place in your Emergency Department during 2004?

2. What do you recall were the intervention strategies used to encourage physicians to use the Canadian CT Head Rule?

3. What do you think were the strengths and weaknesses of these intervention strategies? What do you think worked best?

A. Knowledge

1. Do you use the Canadian Adult CT Head Rule in your practice? What does the rule say? Who does the rule target? What is the clinical condition of the target? What clinical action or behaviour is the rule focused on? Do you recall the steps of the rule?

2. Do you use any other CT Head Rules? What do/does that/those rule(s) say?

(if they state they don't use any particular rule) Is there any reason why you do not use a specific rule to guide management of adults who present to your department with minor head injury?

3. What do you think of the evidence that supports the Canadian Adult CT Head?

B. Skill

4. How do you use the Canadian CT Head rule in your practice? (prompt – What steps do you normally follow when using the rules? Do you ever read or refer back to them? Do you apply them while you are assessing the patient or after)
5. Can you describe a typical clinical situation when you would use the rule?
6. Can you describe a typical clinical situation when you would not use the rule?
7. What skills are needed to use the rule? (prompt – are there any other skills that you need to use the rule)
8. How easy or difficult is it to apply the Canadian Adult CT head rule? (prompt – what makes it easy or difficult for you?)

C. Social/Professional Role

9. Is there anything about belonging to a professional group of emergency room physicians that influences how you use the Canadian CT Head rule? (prompt - do you feel any professional obligation to use evidence based tools such as this CT Head rule)
10. How does using this rule influence your decision making autonomy with this population of patients?
11. Do you see any legal or ethical issues with using this rule?

12. Do your colleagues generally agree with you regarding your use of the Canadian Adult CT Head rule in your department?

D. Beliefs about capabilities

13. How confident do you feel in your ability to apply the Canadian Adult CT Head rule?

14. What problems have you encountered when trying to follow the rule? (prompts – are there specific problems with the rule? Or in your emergency department? Or in your health centre? How can you overcome these problems?)

15. What would help you follow the rule?

E. Beliefs about consequences

16. What do you think are the consequences of using the Canadian Adult CT Head rule? That is, what do you think are the benefits of using the Canadian Adult CT Head rule? (prompts – are there any particular patient benefits, financial benefit, HCP benefits or administration benefits) And is there any harm that can occur as a result of using the Canadian CT Head rule? (prompts – is there any potential harm for the patient, health care professional or the ED?)

17. Do the potential benefits outweigh the potential harms for using the Canadian Adult CT head rule?

F. Motivation and goals

18. How important is the Canadian Adult CT Head rule for managing patients with minor head injury?

19. Would the goal of using the Canadian Adult CT Head rule be incompatible with your usual practice? Why or why not?

20. Are there other rules or guidelines or anything else that conflict with your use of the rule?

21. Are there any incentives for following the CT head rule? What are they?

G. Memory, attention and decision process

22. Is using the Canadian Adult CT Head rule something that you normally do when managing patients with minor head injury?

23. How easy or difficult is it to remember the rule? What would help make it easy to remember the rule?

24. What triggers your decision to use the Canadian Adult CT Head rule? What other factors influence your use of the rule?

25. Are there situations where it is difficult to apply the Canadian Adult CT Head rule? (prompt – can you tell me what it is about these situations that make it difficult to apply the rule)

26. Do you sometimes forget to use the Canadian Adult CT Head rule? When do you forget?

H. Environmental context and resources

27. What factors in your clinical environment influence your decision to use the Canadian CT head rule? (prompt – to what extent do you feel these factors influence your ability to use the rule)
28. What factors outside of your ED environment influence your decision to use the Canadian CT head rule? (Prompt – other departments/services in your health centre or ambulance/EHS)

29. Are there competing tasks or time constraints that influence your decision to use the Canadian CT head rule?

I. Social influences

30. Do other physicians in the ED influence your decision to use the Canadian CT Head Rule? (prompt - How do they influence your use of the rule? To what extent?)

31. Do other physicians outside of the ED influence your decision to use the Canadian CT Head Rule? (prompt - How do they influence your use of the rule? To what extent?)

J. Emotion

32. Does using the Canadian CT Head Rule ever evoke an emotional response in you? (prompt – do you ever feel worried or concerned about using the rule? Do these worries or concerns affect your decision to use the rule?)

33. Do your patients emotions ever affect your decision to use the Canadian CT Head Rule (prompt – what about patients families, do their emotions ever affect your decision to use the rule?)

K. Behavioural regulation

34. What do you think is needed to ensure that you consistently use the Canadian CT Head Rule? (prompt – things specific to you or your department or your health centre)

L. Nature of the behaviour

35. How often do you see adult patients with minor head injury each month/week?

36. Do you usually use the Canadian CT Head Rule when you are managing patients with minor head injury?
